# Supplementary material for: Profiling cell proliferation after whole-genome duplication in human cells
Source: Biol Open. 2026 May 26;15(5):bio062568. doi: 10.1242/bio.062568 (PMC13267767; doi:10.1242/bio.062568)
Supplement: Supplementary information [file biolopen-15-062568-s1.pdf]

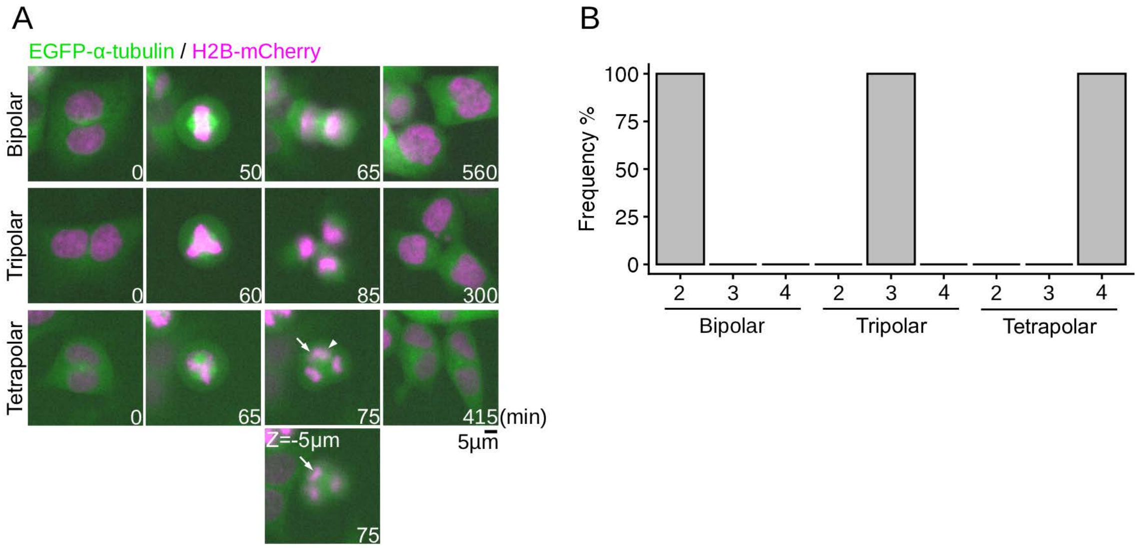

Fig. S1. Correlation between chromosome segregation polarity and daughter nuclear number

(A) Examples of live images of post-WGD cell proliferation (H2B-mCherry and EGFP-α-tubulin HCT116). Images were taken at a 5-min interval. The arrow and arrowhead indicate two distinct chromosome masses detected across different z slices. **(B)** Relationship between chromosome segregation polarity and the total nuclear number in the resulting daughter cells in A. Forty mitotic events from 2 independent experiments are analyzed.

Biology Open • Supplementary information

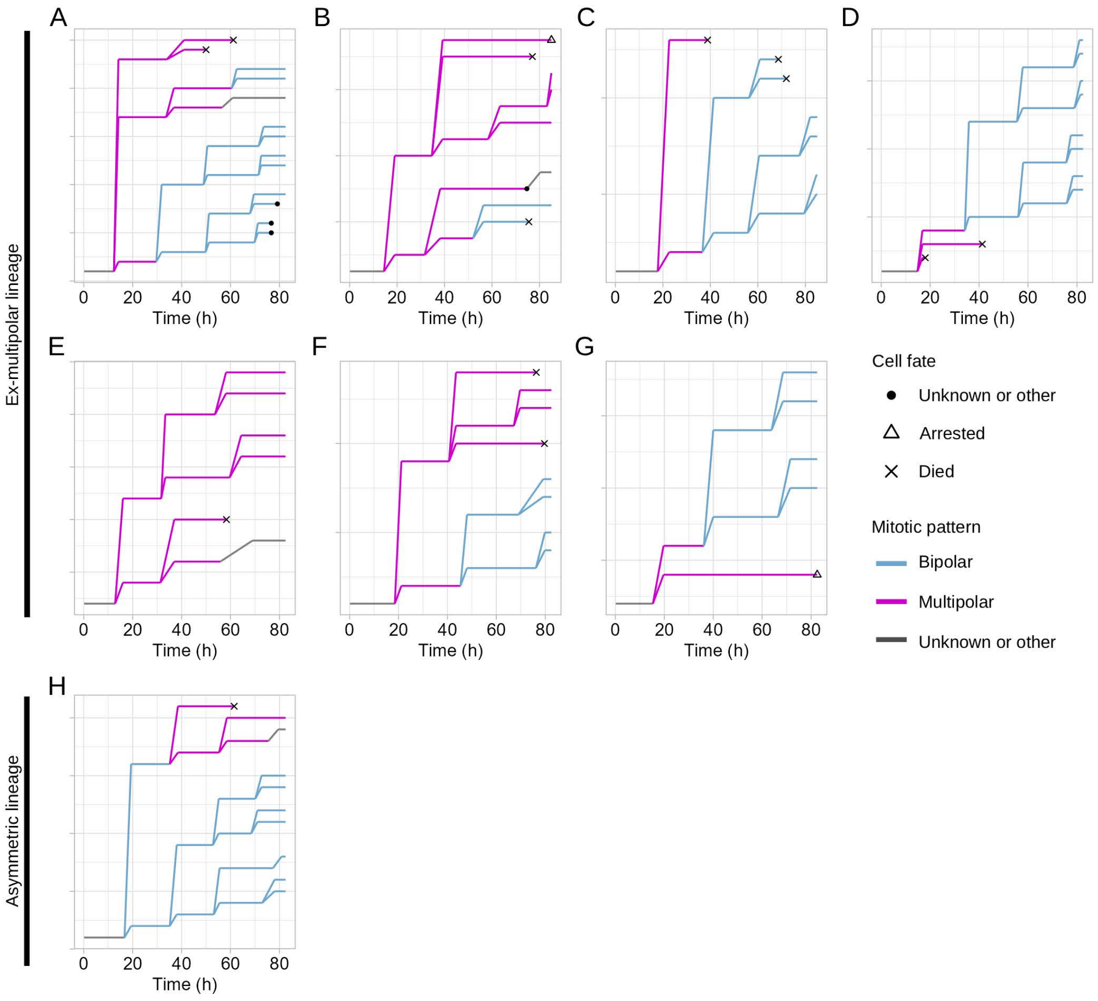

Fig. S2. Mitotic patterns among proliferative post-WGD cell lineages in HeLa cells

(A-H) Tracings of proliferative post-WGD cell lineages throughout the live-imaging. Progenies were color-coded according to mitotic patterns in the previous mitosis. Markers indicated the fates of progenies.

Supplementary material S1

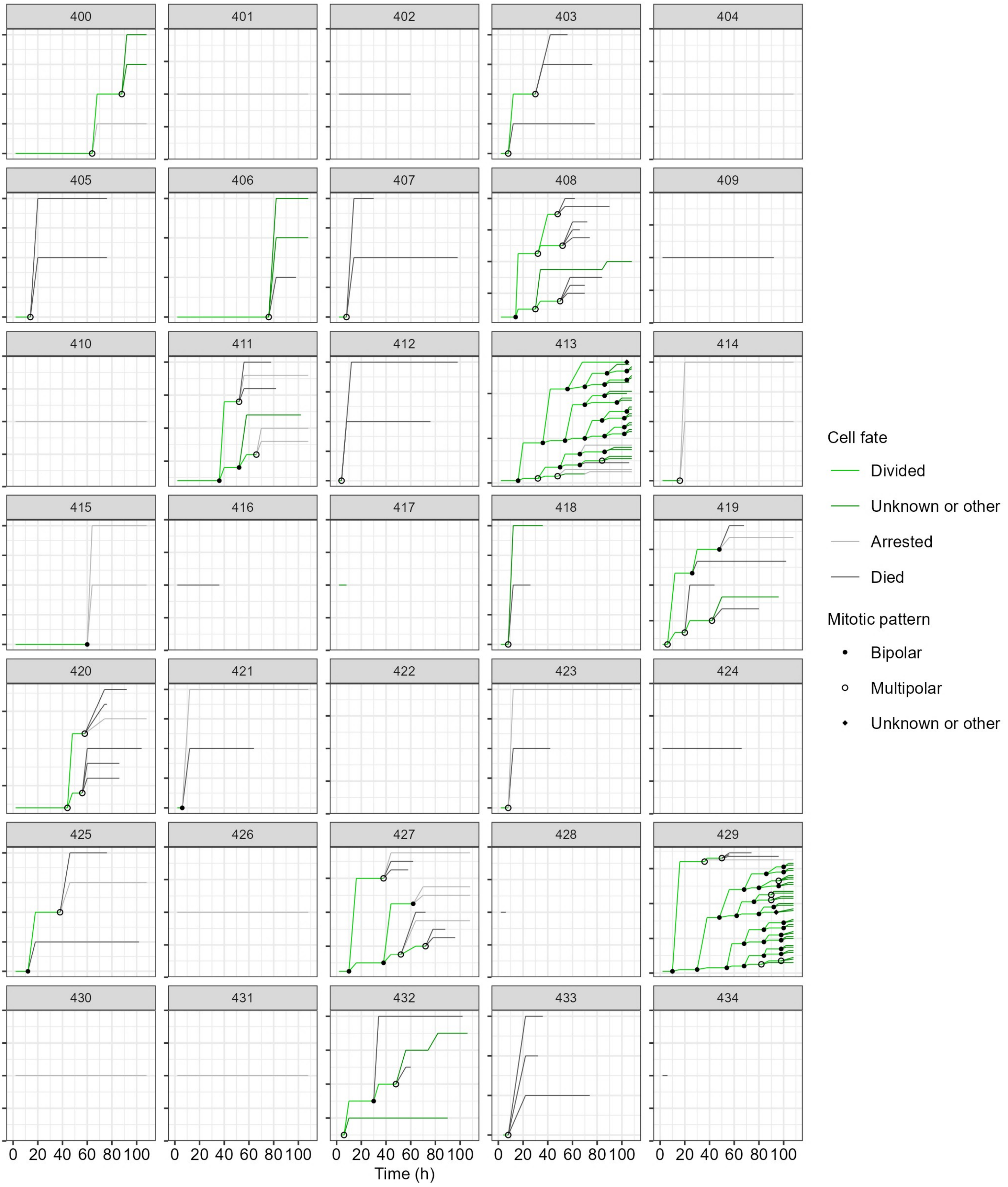

Biology Open • Supplementary information

Supplementary material S1 (continued)

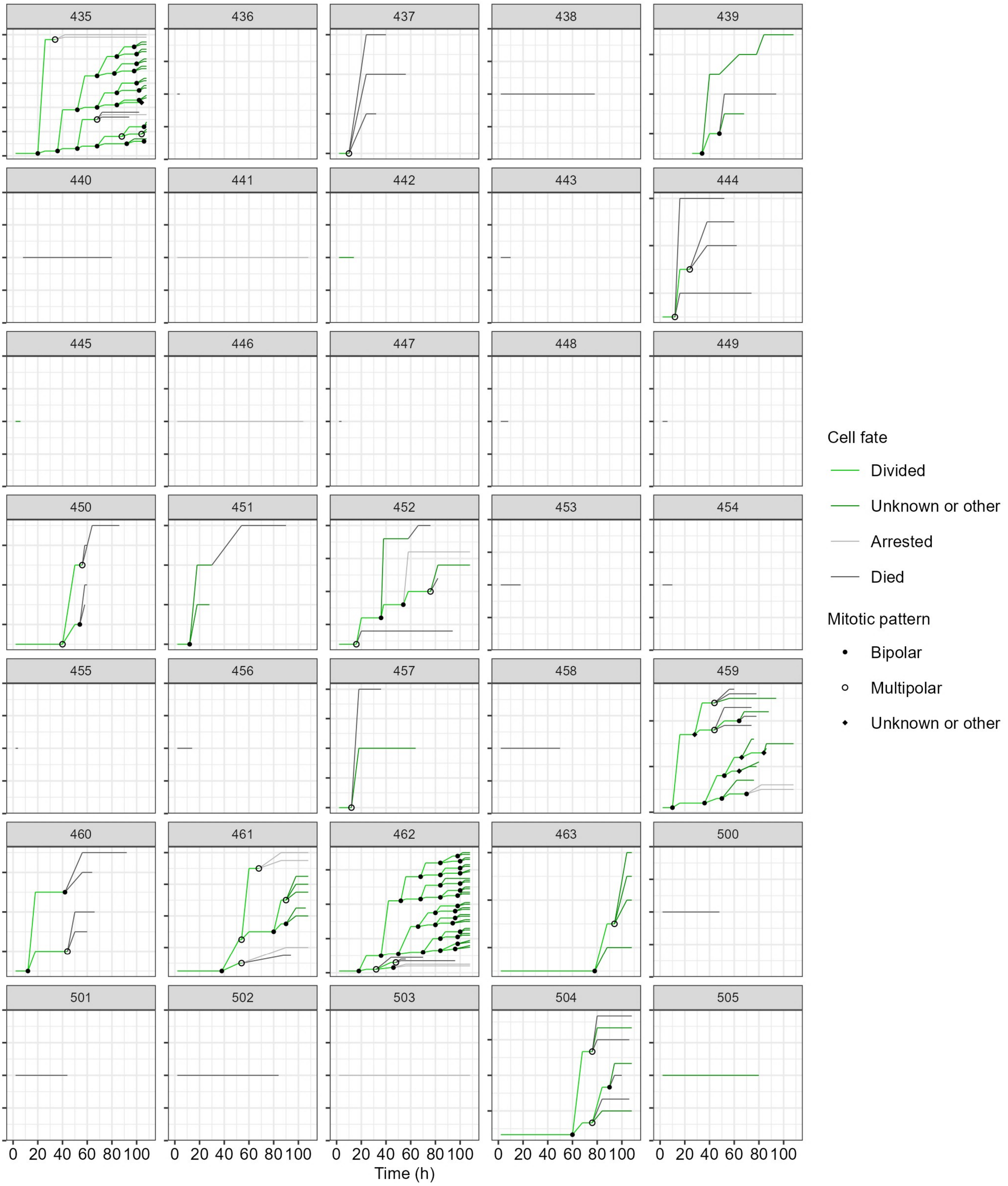

Biology Open • Supplementary information

Supplementary material S1 (continued)

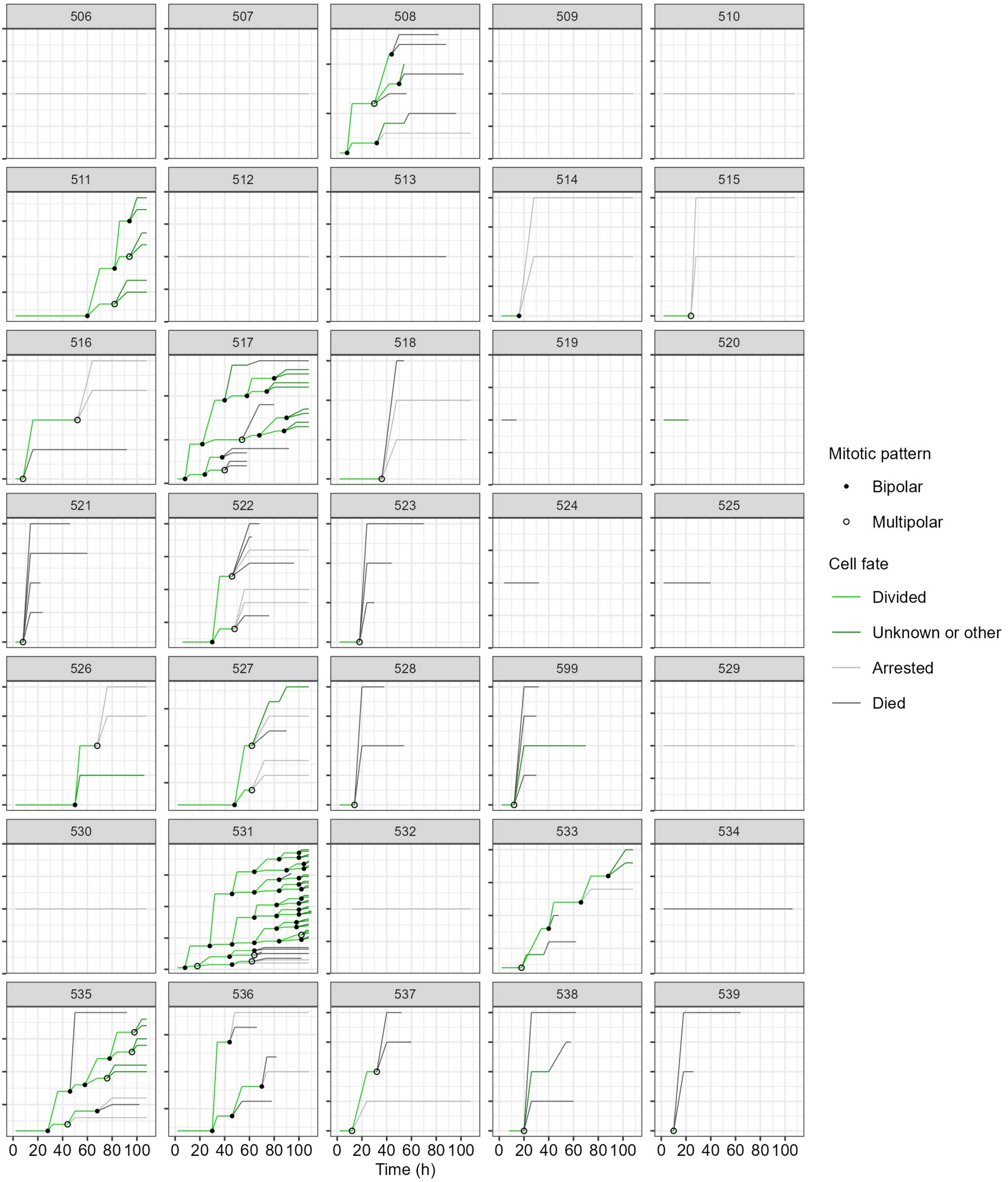

Biology Open • Supplementary information

Supplementary material S1 (continued)

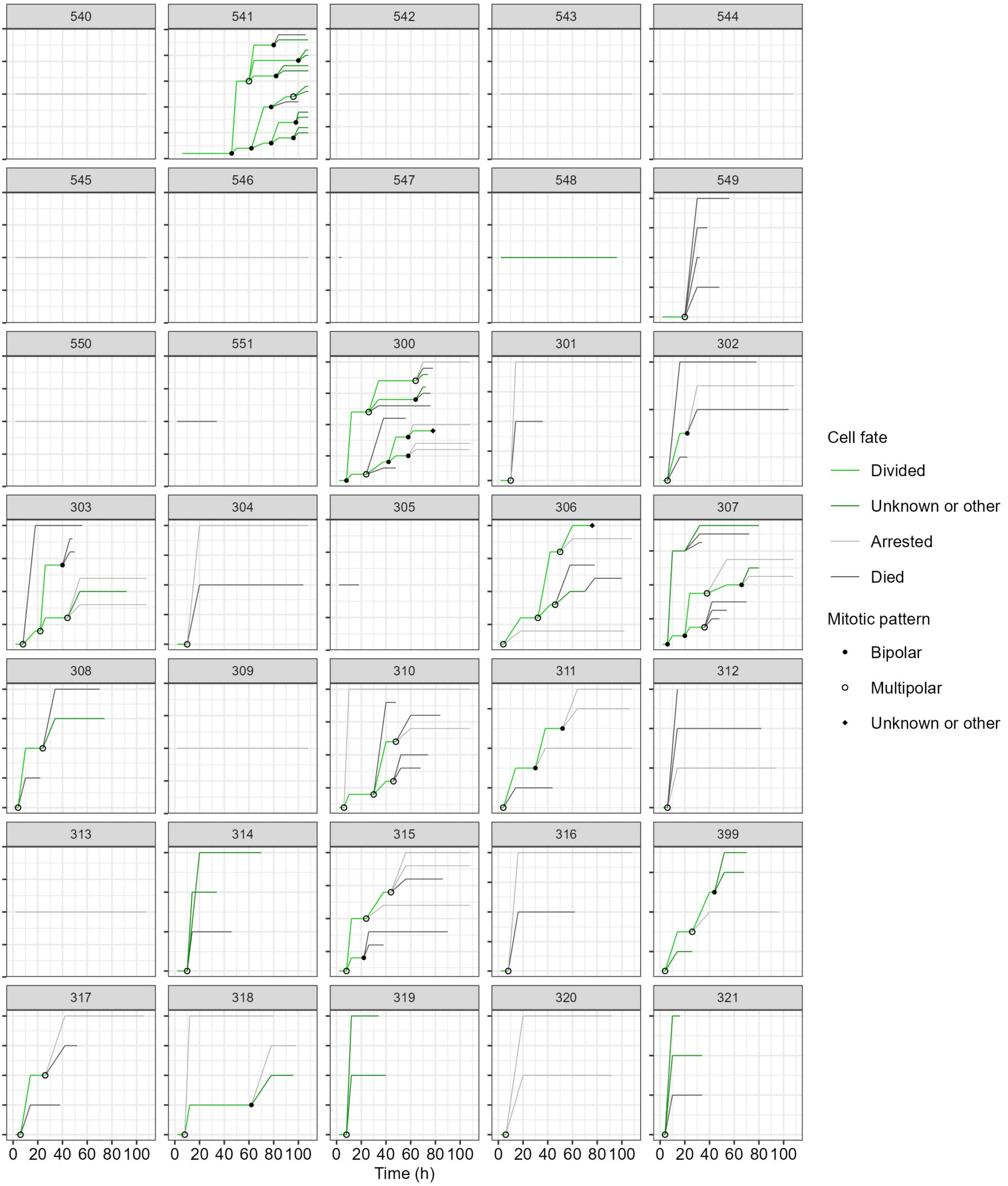

Biology Open • Supplementary information

Supplementary material S1 (continued)

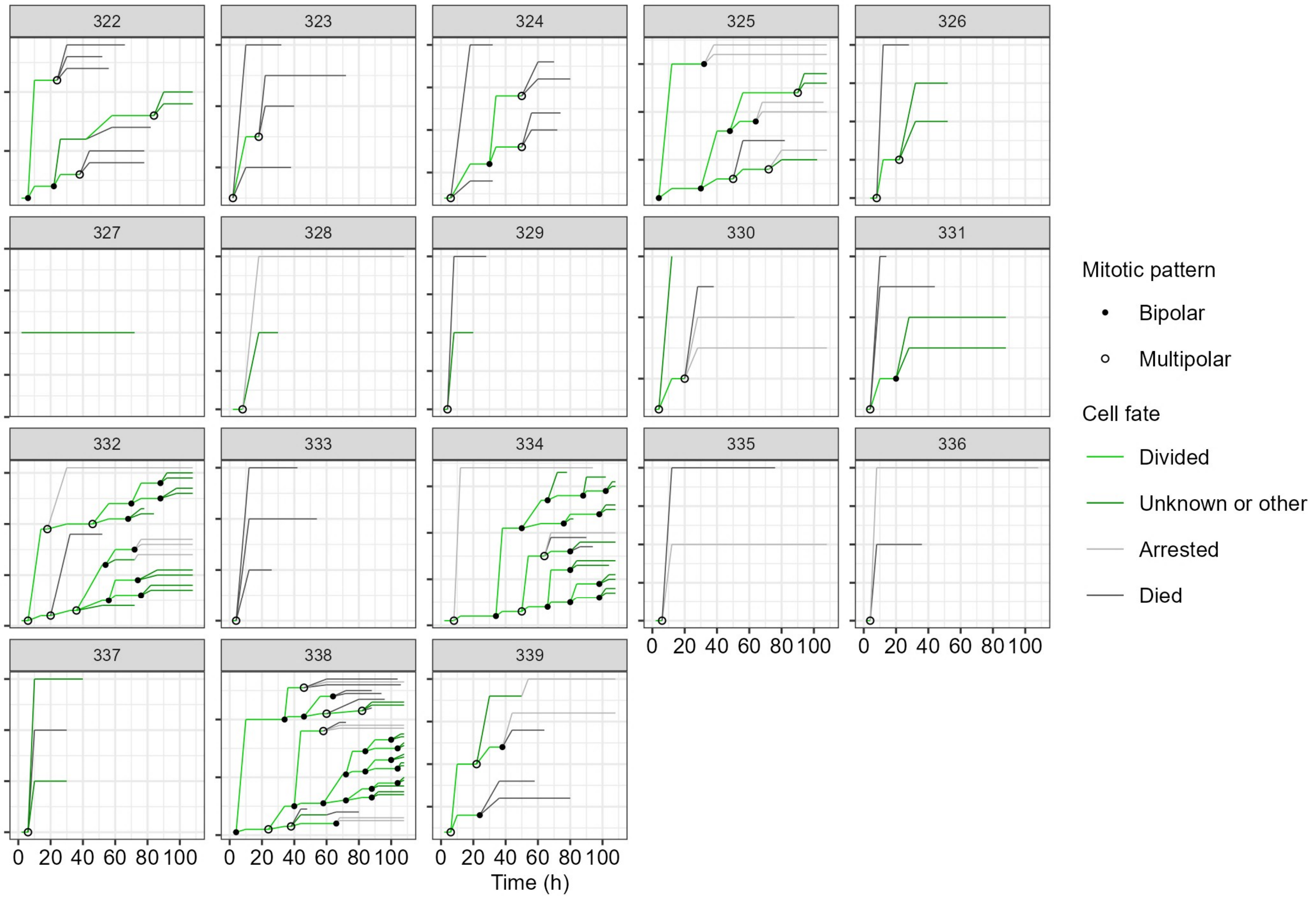

Fig. S3. Tracing of all post-WGD cell lineages

Markers indicated different mitotic patterns. Progenies were color-coded based on their fates.

Biology Open • Supplementary information

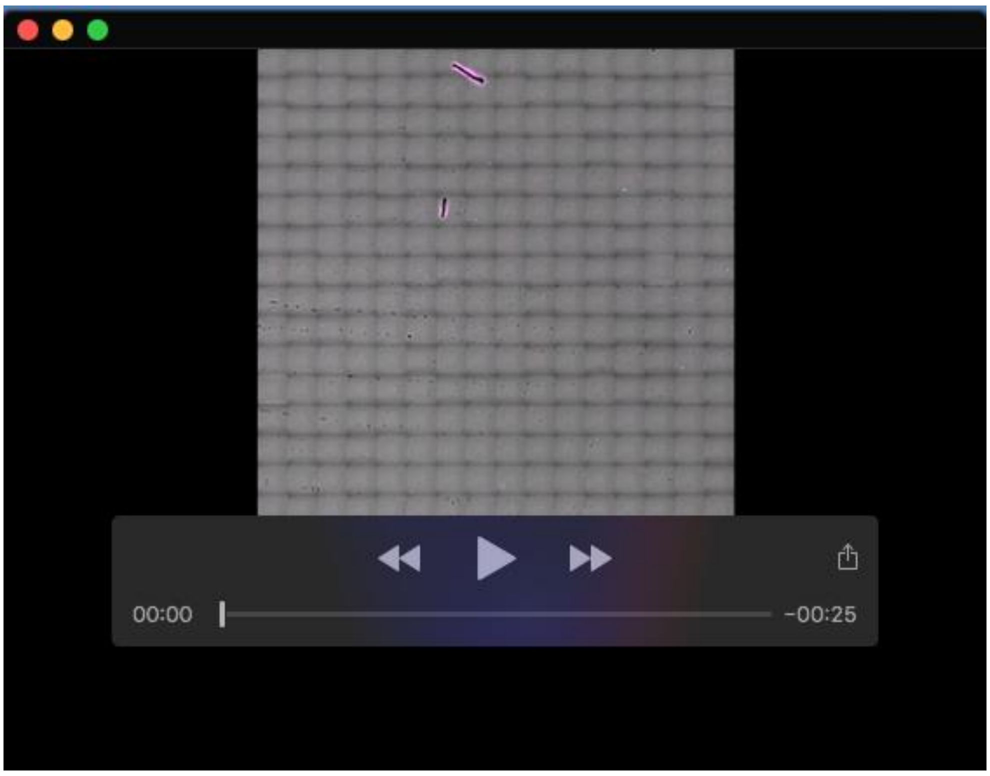

Movie 1. Entire views of all post-WGD cell live images

Live images of post-WGD cell proliferation (2 h per frame). Data from 3 independent experiments are shown in separate movie files. The stitched field of view is 1 cm × 1 cm.

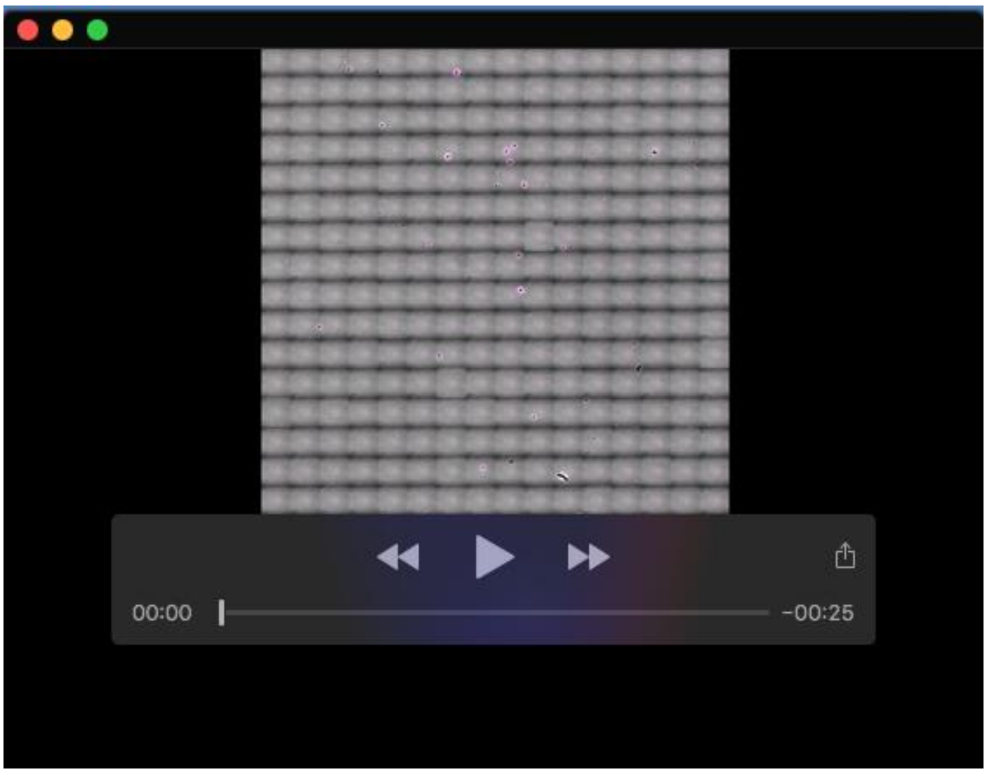

Movie 2. Entire views of all post-WGD cell live images

Live images of post-WGD cell proliferation (2 h per frame). Data from 3 independent experiments are shown in separate movie files. The stitched field of view is 1 cm × 1 cm.

Biology Open • Supplementary information

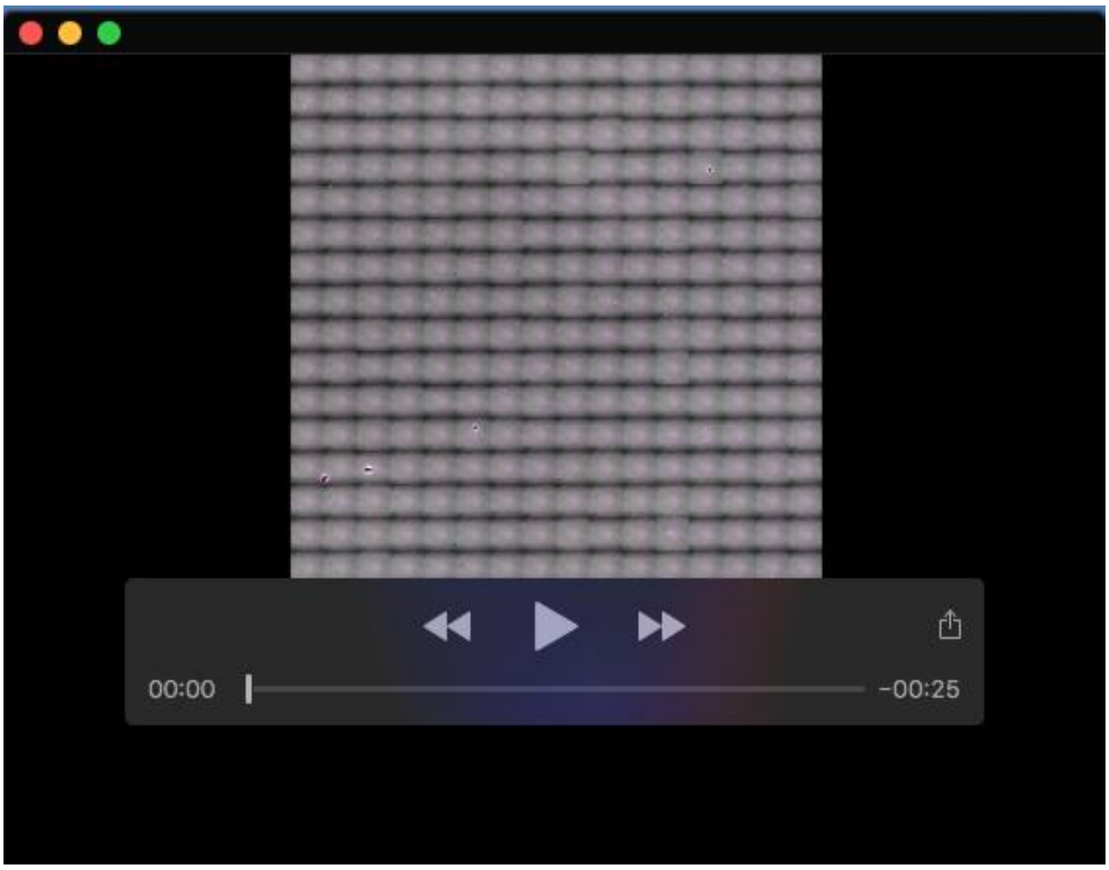

Movie 3. Entire views of all post-WGD cell live images

Live images of post-WGD cell proliferation (2 h per frame). Data from 3 independent experiments are shown in separate movie files. The stitched field of view is 1 cm × 1 cm.

Biology Open • Supplementary information
