# Supplementary material for: Profiling cell proliferation after whole-genome duplication in human cells
Source: Biol Open. 2026 May 26;15(5):bio062568. doi: 10.1242/bio.062568 (PMC13267767; doi:10.1242/bio.062568)
Supplement: Supplementary file 1 [file biolopen-15-062568_review_history.pdf]

Profiling cell proliferation after whole-genome duplication in human cells

Guang Yang, Masaya Inoko, Kaito Ogura, Sumire Ishida-Ishihara, Yuki Tsukada, Akira Funahashi, Masanao Sato and Ryota Uehara
10.1242/bio.062568

Editor: Alissa Armstrong

Review timeline

Original submission:	13 March 2026
Editorial decision:	20 March 2026
First revision received:	25 April 2026
Accepted:	30 April 2026

Original submission

First decision letter

MS ID#: bio.062568

MS Title: Profiling cell proliferation after whole-genome duplication in human cells

Authors: Guang Yang, Masaya Inoko, Kaito Ogura, Sumire Ishida-Ishihara, Yuki Tsukada, Akira Funahashi, Masanao Sato and Ryota Uehara

I have now reached a decision on the above manuscript.

The reviewer reports are shown at the bottom of this email.

As you will see, at least one reviewer raised a substantial criticism that prevents me from accepting the paper at this stage. Generally speaking, however, both reviewers thought the study was carefully conducted and well written.

They suggest, however, that a revised version might prove acceptable, if you can address their concerns. If you think that you can deal satisfactorily with the criticisms on revision, I would be pleased to see a revised manuscript. We would then return it to the reviewers.

At this stage, we also ask you to ensure your manuscript complies with our formatting guidelines. Provided you are able to fully address the referees' comments, we are positive about publication of your paper (we accept over 95% of revision submissions) and therefore hope you won't mind any extra work involved in reformatting your manuscript at this point.

Please upload both a 'clean' version of your Word file, along with a highlighted version clearly showing where you have made changes in the revised manuscript. Please avoid using 'Track changes' in Word files as these are lost in PDF conversion.

I should be grateful if you would also provide a point-by-point response detailing how you have dealt with the points raised by the reviewers in the 'Response to Reviewers' box. Please attend to all of the reviewers' comments. If you do not agree with any of their criticisms or suggestions please explain clearly why this is so.

© 2026. Published by The Company of Biologists under the terms of the Creative Commons Attribution License (https://creativecommons.org/licenses/by/4.0/).

Reviewer 1

Comments for the author

This research article is an original and novel study focus on the identification the determinants behind the progression/survival of cells following induced whole genome duplication (WGD). As a model, authors limited their study to a descriptive analysis of HCT116 cells forced to go through WGD using cytochalasin B. Overall, the manuscript is well written, the goal is clear and data looks robust. The experimental design and results provide sufficient novelty for its publication. However, I recommend the authors to consider the next changes before the acceptance of the manuscript.

Major points:

1. The work uniquely employ HCT116 cells, a colon cancer-derived cell line. I recommend authors to consider the use of other different cancer cell lines to test whether the main observations could be extended. I also encourage the authors to test, at least the main observations, in diploid non-transformed cells like RPE cells. Results will inform whether transformed and non-transformed cells display a similar/different behavior following WGD.

Minor points:

1. Authors describe that multipolar chromosome segregation correlates with the non-proliferative fate of descendent cells (lines 55-59). Data support this correlation although it is not clear to me the causality relationship. Authors must review and reword this section accordingly or provide further mechanistic data supporting the original idea.

2. Figure 3B and C show the quantification of the chromosome distribution ratio in daughter cells. I recommend the authors to represent this data in superplots (Lord 2020 JCB) where measurements from each replicate share the same color. These plots also show the average for each replicate and provide a better indication about the consistence/variance between replicates.

Reviewer 2

Comments for the author

In their manuscript "Profiling cell proliferation after whole-genome duplication in human cells", Yang et al. used live-cell imaging to track mitotic patterns and cell fate after whole-genome duplication (WGD) over multiple generations. Multipolar chromosome segregation is suggested to be a key event that limits the proliferative capacity of WGD progeny.
The text is well written and the figures are clear allowing for a quick grasp of the main findings. However, I have a major concern with the way of how multipolar chromosome segregation was detected in these experiments. The authors used live-cell imaging with a time lapse of 2 hours, but chromosome segregation typically occurs within 30 minutes. Do the analyses only include cells that were actually captured during anaphase? Figure 1 shows examples of progeny from putative bipolar or multipolar chromosome segregation, is this just an assumption based on nuclear morphology? Moreover, the mitotic spindle or centrosomes are not labelled in these experiments and it might be difficult to detect abnormal spindle configurations by solely looking at H2B. In line with this, centrosome "elimination" in proliferative lineages remains purely speculative.

There are some additional minor issues:
-page 2, line 33: "bioprocesses" should be replaced with biological processes or similar (see also page 13, line 4)
-page 7, line 37-42: "Interestingly, in both proliferative and non-proliferative lineage groups, cell cycle length after the second generation became drastically shorter than that in the first generation". When looking at the respective figure panel, it appears that cell cycle length was rather drastically shorter after the first generation (maybe specify the timings in the text as it is hard to retrieve this information from the graph). However, this would not be that surprising as the first spindle assembly was taking place after Nocodazole wash-out which is known to significantly prolong this process. Moreover, this method drastically increases the frequency of lagging chromosomes that potentially cause the formation of micronuclei. Do the analyses only account for

© 2026. Published by The Company of Biologists under the terms of the Creative Commons Attribution License (https://creativecommons.org/licenses/by/4.0/).

lineages that originate from a binucleated cell? Otherwise, this factor might easily influence cell fate and should be discussed.
-page 14, "cell culture": Did the authors use one clonal line for their experiments or several (if the latter is true, this might influence cell fate independent of the chromosome segregation pattern)? Replating of cells after shake-off should be mentioned
- the methods section should include a short description of how the nuclear ratio was determined (sum intensity of the entire nucleus?)
-page 14, live cell imaging: how many z-stacks were used (maybe also include the time interval here again)
-page 17, line 25: what means "makers" in this context? (also at other places in the text)
-Fig. 2B: specify what the boxes/bars/lines indicate
-Fig. 2C: add "(%)" to the y-achsis
-Fig. 3B: Does this graph also include cells that died after cell division? If so, would the distribution on both sides of the mean in the condition "multipolar 2" not look identical?

Reviewer's Responses to Questions

Experimental quality
Does each figure have the proper controls?
If 'No', please indicate reasons in Comments for Author box below.
Reviewer #1:
- Yes

Reviewer #2:
- No

Were the data analyzed using appropriate statistical tests?
If 'No', please indicate reasons in Comments for Author box below.
Reviewer #1:
- Yes

Reviewer #2:
- Yes

Reproducibility
Were experiments performed using adequate number of biological replicates?
If 'No', please indicate reasons in Comments for Author box below.
Reviewer #1:
- Yes

Reviewer #2:
- Yes

Does the methods section provide sufficient detail to permit reproducibility?
If 'No', please indicate reasons in Comments for Author box below.
Reviewer #1:
- Yes

Reviewer #2:
- Yes

Completeness
Are the manuscript's conclusions supported by the data?
If 'No', please indicate reasons in Comments for Author box below.
Reviewer #1:

© 2026. Published by The Company of Biologists under the terms of the Creative Commons Attribution License (https://creativecommons.org/licenses/by/4.0/).

- Yes

Reviewer #2:
- No

Scholarship
Do the authors cite and discuss the merits of data that would argue for and against their conclusion?
If 'No', please indicate reasons in Comments for Author box below.
Reviewer #1:
- Yes

Reviewer #2:
- No

Does the manuscript title & abstract accurately reflect the contents of the manuscript, without hyperbole?
If 'No', please indicate reasons in Comments for Author box below.
Reviewer #1:
- Yes

Reviewer #2:
- Yes

First revision
Dear Prof. Alissa Richmond Armstrong,

We are sincerely grateful to you for handling our manuscript entitled "Profiling cell proliferation after whole-genome duplication in human cells" (MS ID# bio.062568)

Editor Comments:

As you will see, at least one reviewer raised a substantial criticism that prevents me from accepting the paper at this stage. Generally speaking, however, both reviewers thought the study was carefully conducted and well written.
They suggest, however, that a revised version might prove acceptable, if you can address their concerns. If you think that you can deal satisfactorily with the criticisms on revision, I would be pleased to see a revised manuscript. We would then return it to the reviewers.

We would like to express our sincere gratitude to you for handling our manuscript. We have addressed all the reviewers' points with new experiments, analyses, and substantial edits to the manuscript. Point-by-point responses are in the following sections of this letter.

In particular, we have strengthened the data or refined our interpretations regarding the following key points, which we believe have solidified the conclusions or improved the rationality of our study:
1. We added new post-WGD lineage analyses in the HeLa cancer cell line. These new analyses provided information on the generality and diversity of the findings in the original manuscript. While an additional trial to trace post-WGD proliferation in hTER-RPE1 cells was precluded due to stringent cell cycle arrest, we believe this result also provides insight into diversity in cellular responses after acute WGD induction.
2. We conducted live imaging at higher temporal resolution to verify our approach for determining chromosome segregation polarity based on the total number of daughter nuclei.

© 2026. Published by The Company of Biologists under the terms of the Creative Commons Attribution License (https://creativecommons.org/licenses/by/4.0/).

3. We refined several quantifications and explanations of the methodology to improve the manuscript's readability and repeatability.

We hope you will find this revised manuscript significantly improved and suitable for publication in Biology Open. We thank you again for considering our manuscript for publication. I look forward to hearing from you.

Comments from the Reviewers:

Response to the comments by Reviewer #1:

We are grateful to Reviewer #1 for the constructive suggestions to improve the generality and diversity of the findings across different cellular backgrounds. We have now addressed all comments raised.

Major points:

1. The work uniquely employ HCT116 cells, a colon cancer-derived cell line. I recommend authors to consider the use of other different cancer cell lines to test whether the main observations could be extended. I also encourage the authors to test, at least the main observations, in diploid non-transformed cells like RPE cells. Results will inform whether transformed and non-transformed cells display a similar/different behavior following WGD.

Rev#1-1:
As another cancer cell model, we newly conducted live imaging analysis in WGD-induced HeLa cells. The newly conducted lineage tracing revealed prominent similarities in mitotic patterns after WGD between HCT116 and HeLa: "Asymmetric" and "Ex-multipolar" were the two major lineage types observed in these lines. Meanwhile, the most frequent lineage type was different between them ("Ex-multipolar" lineages were most frequent in HeLa). The new results demonstrate common and diverse aspects of post-WGD cell proliferation across different cell backgrounds. We provided the new data in HeLa cells as Fig. S2 (the sentences starting with "*To gain insights into the generality or diversity..*").

Although we also attempted to test post-WGD proliferation in hTERT-RPE1 cells, none of the WGD-induced cells (48 cells from 3 independent experiments) underwent the first mitosis at least 28 h after WGD induction. To overcome this issue and test the post-WGD proliferation dynamics in natural, non-perturbed conditions in non-cancer backgrounds, it would be necessary to comprehensively screen for non-cancer cells that naturally exhibit less stringent post-WGD cell cycle arrest control; however, we judged that this was beyond the scope of the experiment within the given revision period. We added a discussion on this topic in the revised manuscript (the sentences starting with "*Although we also attempted to investigate post-WGD lineages…*").

Minor points:

1. Authors describe that multipolar chromosome segregation correlates with the non-proliferative fate of descendent cells (lines 55-59). Data support this correlation although it is not clear to me the causality relationship. Authors must review and reword this section accordingly or provide further mechanistic data supporting the original idea.

Rev#1-2:
We rewrote the manuscript to clearly state that the data support only a correlation and that future studies are required to address causality (The sentences starting from "*This data demonstrates a correlation between ….*"). We also rewrote the manuscript to use more conservative wording regarding the possible relationship between multipolar chromosome segregation and post-WGD cell fates.

2. Figure 3B and C show the quantification of the chromosome distribution ratio in daughter cells. I recommend the authors to represent this data in superplots (Lord 2020 JCB) where measurements from each replicate share the same color. These plots also show the average for each replicate and provide a better indication about the consistence/variance between replicates.

© 2026. Published by The Company of Biologists under the terms of the Creative Commons Attribution License (https://creativecommons.org/licenses/by/4.0/).

Rev#1-3:
We reanalyzed the data accordingly. Please note that, due to the preference in the script used for graph drawing, the measurements from each replicate were indicated by the same marker shapes instead of colors. The new data also support the conclusion.

Response to the comments by Reviewer #2:

We are grateful to Reviewer #2 for the important critiques, especially on the accuracy of detection of multipolar chromosome segregation events. We have now addressed all comments raised.

The text is well written and the figures are clear allowing for a quick grasp of the main findings. However, I have a major concern with the way of how multipolar chromosome segregation was detected in these experiments. The authors used live-cell imaging with a time lapse of 2 hours, but chromosome segregation typically occurs within 30 minutes. Do the analyses only include cells that were actually captured during anaphase? Figure 1 shows examples of progeny from putative bipolar or multipolar chromosome segregation, is this just an assumption based on nuclear morphology? Moreover, the mitotic spindle or centrosomes are not labelled in these experiments and it might be difficult to detect abnormal spindle configurations by solely looking at H2B. In line with this, centrosome "elimination" in proliferative lineages remains purely speculative.

Rev#2-1:
As noted by Reviewer #2, we were unable to capture anaphase events in some cases. We determined the polarity of chromosome segregation based on the total number of nuclei in the resulting daughter cells. To validate this approach, we conducted a new 5-min interval live-imaging experiment using WGD-induced HCT116 cells expressing EGFP-α-tubulin and H2B-mCherry. We then quantitatively analyzed the relationship between chromosome segregation polarity and the number of nuclei in the resulting daughters. We confirmed that chromosome segregation polarity and the total number of nuclei matched 100%. We rewrote the manuscript to clearly describe this issue with the presentation of the above pilot experimental results in Fig. S1 (sentences starting with "*Note that, with a 2-h interval, anaphase chromosome segregation…*").

With the spatial resolution of the microscopy used here, it was impossible to visualize the centrosomes (please note that higher resolution, enabling centrosome visualization, causes severe phototoxicity, preventing long-term cell survival). We rewrote the manuscript to discuss this point and to emphasize that any thoughts on centrosome dynamics here are speculative and require careful future investigation (sentences starting with "*However, with the microscopy's spatial resolution..*").

There are some additional minor issues:
-page 2, line 33: "bioprocesses" should be replaced with biological processes or similar (see also page 13, line 4)

Rev#2-2:
We rewrote the corresponding parts of the manuscript as per the suggestion.

-page 7, line 37-42: "Interestingly, in both proliferative and non-proliferative lineage groups, cell cycle length after the second generation became drastically shorter than that in the first generation". When looking at the respective figure panel, it appears that cell cycle length was rather drastically shorter after the first generation (maybe specify the timings in the text as it is hard to retrieve this information from the graph). However, this would not be that surprising as the first spindle assembly was taking place after Nocodazole wash-out which is known to significantly prolong this process. Moreover, this method drastically increases the frequency of lagging chromosomes that potentially cause the formation of micronuclei. Do the analyses only account for lineages that originate from a binucleated cell? Otherwise, this factor might easily influence cell fate and should be discussed.

Rev#2-3:
We agree that the cell cycle difference was most evident between the first and second cell cycles, and we should have described it as "after the first cell cycle," rather than "after the second cell

© 2026. Published by The Company of Biologists under the terms of the Creative Commons Attribution License (https://creativecommons.org/licenses/by/4.0/).

cycle." Per the comments, we added a discussion of the possible effects of nocodazole treatment on the dynamics of the first cell cycle (sentences starting with "*This difference in cell cycle length could..*").

In this experiment, we only analyzed binucleated cells: We realized that quantification of the first cell cycle length in non-WGD-induced diploid inner control cells is not applicable, since it is impossible to accurately specify when these diploids went into the cell cycle before the initiation of the live imaging (please note that most of these cells avoided WGD-induction because they were not in mitosis when cytochalasin was administrated). Therefore, we described the limitations of our interpretation on this issue in the manuscript.

-page 14, "cell culture": Did the authors use one clonal line for their experiments or several (if the latter is true, this might influence cell fate independent of the chromosome segregation pattern)? Replating of cells after shake-off should be mentioned

Rev#2-4:
For the main live-imaging experiments, we used a single clonal diploid H2B-mCherry HCT116 line as the source of WGD induction. Please note that the new experiments associated with Rev#2-1 were conducted using another single clonal H2B-mCherry/ EGFP-α-tubulin HCT116 line.

Thank you for pointing out the lack of information on replating. We add this information to the corresponding section of the "Cell culture" section.

- the methods section should include a short description of how the nuclear ratio was determined (sum intensity of the entire nucleus?)

Rev#2-5:
We added a detailed description of the quantification in the Materials and methods section (sentences starting with "*To obtain the chromosome distribution ratio, the total..*").

-page 14, live cell imaging: how many z-stacks were used (maybe also include the time interval here again)

Rev#2-6:
Per the comment, we added the information to the relevant section of the Materials and methods.

-page 17, line 25: what means "makers" in this context? (also at other places in the text)

Rev#2-7:
These were typos of "markers." We amended these errors. We apologize for the confusion caused by these errors.

-Fig. 2B: specify what the boxes/bars/lines indicate

Rev#2-8:
We added the information to the corresponding figure legend.

-Fig. 2C: add "(%)" to the y-achsis

Rev#2-9:
Thank you for the correction. We added % to the graph legend.

-Fig. 3B: Does this graph also include cells that died after cell division? If so, would the distribution on both sides of the mean in the condition "multipolar 2" not look identical?

Rev#2-10:
This graph includes cells that died after cell division. However, in all cases, subsequent cell death occurred later than the time frame at which nuclear H2B-mCherry signals were quantified. Therefore, we believe nuclear events associated with the progression of cell death would not have affected the quantification. We added a note on this issue in the corresponding figure legend.

© 2026. Published by The Company of Biologists under the terms of the Creative Commons Attribution License (https://creativecommons.org/licenses/by/4.0/).

Second decision letter

MS ID#: bio.062568R1

MS Title: Profiling cell proliferation after whole-genome duplication in human cells

Authors: Guang Yang, Masaya Inoko, Kaito Ogura, Sumire Ishida-Ishihara, Yuki Tsukada, Akira Funahashi, Masanao Sato and Ryota Uehara

I am happy to tell you that your manuscript has been accepted for publication in Biology Open, pending our standard publication integrity checks. It was accepted on 30th April 2026.

© 2026. Published by The Company of Biologists under the terms of the Creative Commons Attribution License (https://creativecommons.org/licenses/by/4.0/).
